# Hand Mycetoma: The Mycetoma Research Centre Experience and Literature Review

**DOI:** 10.1371/journal.pntd.0004886

**Published:** 2016-08-02

**Authors:** Rowa Fathelrahman Omer, Nancy Seif EL Din, Fadwa Awad Abdel Rahim, Ahmed Hassan Fahal

**Affiliations:** The Mycetoma Research Centre, University of Khartoum, Khartoum, Sudan; University of Tennessee, UNITED STATES

## Abstract

Mycetoma is a devastating, neglected tropical disease characterised by extensive tissue involvement resulting in destruction, deformities and disabilities in the affected patients. The hand is commonly affected by mycetoma thus compromises its functionality and hinder the patient’s daily activities of living. In this communication, we report on 533 patients with hand mycetoma managed over a period of 24 years at the Mycetoma Research Centre, University of Khartoum, Khartoum, Sudan. Eumycetoma was the commonest type of mycetoma (83.3%) encountered. Males were predominately affected (69.2%) with a sex ratio of 2.2:1. The majority of the patients (84%) were young adult below the age of 40 years old at presentation. The generality of patients (86.4%) were from the Sudan mycetoma belt. Children and adolescents (28.1%), farmers (18.2%) and workers (17.4%) were more frequently affected. The majority of patients (67.4%) had disease duration of less than 5 years at presentation. The study, did not document significant history of local trauma, familial tendency, concomitant medical diseases or other predisposing cause for mycetoma in this population. Pain (23.1%) was not a disease feature in this series and 52% of patients had past surgery for mycetoma and recurrence. The right hand was affected most (60.4%), and 64% of them had small lesion at presentation. Conventional x-ray was only helpful in patients with advanced disease and the MRI accurately determined the disease extension. Cytological smears, surgical biopsies histopathological examination and grains culture were the principal diagnostic tools for causative organisms’ identification. In the present series it was difficult to determine the treatment outcome due to high patients follow up dropout.

## Author Summary

Hand mycetoma is a serious condition due to the complexity of the hand anatomy and functionality. The disease and the surgical intervention can both produce distortions, deformities and disabilities which interfere with patient daily activities of living. Thus producing huge burden on the patients, the families and inflicts numerous socio-economic bearings on the communities in endemic regions.

The present study, identified that, most patients were young adult males, of poor socioeconomic status and from rural areas of the Sudan. Compared with patients with mycetoma in other parts of the body, they presented rather early having noticed the lesion earlier because they frequently wash and examine their hands. No predisposing factors were evidenced in the study. Several diagnostic tools and techniques were required to establish the diagnosis of mycetoma in these patients and hence there is a need for a simple, affordable, field friendly diagnostic test which is of reasonable sensitivity and specificity. The treatment is prolonged and in most cases the outcome was unsatisfactory, thus new novel treatment is desirable. Until that time, a well-structured health education programmes are required to encourage early presentation and treatment compliance.

## Introduction

Mycetoma is a highly devastating and distressing neglected tropical disease characterized by painless subcutaneous masses with multiple sinuses draining pus and grains [1, 2]. The disease leads to massive deformities, disabilities with severe adverse bearings on the affected patients [3, 4, 5]. The aetiological causative agents are either fungi or actinomycetes and thus it is classified as eumycetoma and actinomycetoma. *Madurella mycetomatis* is the commonest eumycetoma causative agent, while *Streptomyces somaliensis* and *Nocardia spp*. are the common actinomycetes causing actinomycetoma [6, 7, 8]. This chronic infection is believed to be caused by subcutaneous inoculation of the causative organisms through minor injuries which remain inactive for years. The infection eventually spread to involve the skin and the deep structures, resulting in destruction, deformity and loss of function, occasionally it can be fatal [9, 10]. It has enormous socioeconomic effects on the affected communities [11, 12].

The foot and hand are the most frequently affected sites accounting for 82% of cases [13,14], other parts of the body may be involved such as the knee, arm, leg, head and neck, thigh and perineum. No age is exempted in mycetoma; however, it occurs more frequently in young adult men in the age range 15–30 years and almost 30% of reported patients are children [15].

In order to provide proper treatment, it is important to identify the causative agent and the disease extension and this can be achieved by a battery of investigations [16,17,18,19,20]. Treatment of mycetoma is unsatisfactory and recurrence is common [21,22]. A combination of surgical excisions and antifungal agents for eumycetoma and antibiotics combination for actinomycetoma are currently the recommended standard treatment [23,24,25,26,27]. Mycetoma patients need close and continuous clinical follow up to detect recurrence which is common problem.

## Materials and Methods

At the Mycetoma Research Centre (MRC), University of Khartoum, Khartoum, Sudan, this descriptive, retrospective, chart-review based case series study was conducted. The electronic patients’ records of 533 patients with confirmed hand mycetoma seen in the period between 1991 and 2015 were carefully and meticulously reviewed.

The diagnosis of mycetoma was confirmed by careful interview, meticulous clinical examinations and several diagnostic tools and techniques. These diagnostic tools and techniques included fine needle aspiration for cytology (FNAC), histopathological examination of surgical biopsies using different staining techniques. Grains were cultured in various media and that included modified Sabouraud agar supplemented with 0.5% yeast extract, Blood, brain-heart infusion and Löwenstein media. Different imaging techniques and these included conventional x-ray in at least two views: anterio-posterior and lateral, lesion ultrasound examination were done and in some patients MRI and CT scan were performed.

### Ethical Statement

The study ethical clearance was obtained from Soba University Hospital Ethical Committee, it waived the need for informed patients consent.

### Statistical Analysis

The data was managed by SPSS computer programme. Data was summarized as percentages for categorical variables and mean for continuous variables.

## Results

The study included 533 patients with confirmed hand mycetoma seen in the period 1991 and 2015, comprise 7.4% of the total patients seen during that study period. There were 369 males (69.2%) and 164 females (30.8%) with a sex ratio of 2.2:1. Most of the patients had eumycetoma 444 (83.3%) and actinomycetoma was seen in 89 patients (16.7%) (Table 1).

**Table 1:** The demographic characteristics of the studied population.

The Demographic Characteristics	No.	%
**Sex**		
Male	369	69.2
Female	164	30.8
Age in years		
<20	158	29.6
20–40	290	54.4
40+	84	15.8
Occupation		
House wife	93	17.4
Worker	93	17.4
Farmer	97	18.2
Clerk	2	0.4
Jobless	54	10.1
Children & adolescents	150	28.1
Employee	5	.9
Others	37	6.9
Missing	2	0.4
Duration		
<5	359	67.4
5–10	124	23.3
>10	48	9.0
Total	531	99.6
Missing	2	.4
Yes	123	23.1
No	390	73.2
Sometimes	10	1.9
Missing	10	1.9
Trauma		
Yes	104	19.5
No	360	67.5
Not sure	61	11.4
Missing	8	1.5
Family history		
Yes	59	11.1
No	407	76.4
Missing	65	12.2
Medical problem		
Yes	18	3.4
No	512	96.1
Missing	3	.6
0	256	48.0
1	190	35.6
2	51	9.6
3	16	3.0
4	19	3.6
Missing	1	.2

The majority of the studied patients, 449 (84.2%) were below 40 years old at presentation, 158 patients (23.8%) were below 20 years old and only 22 patients (4.1%) were above 60 years old.

Children and adolescents were affected most; 150 (28.1%), followed by farmers 97 (18.2%) and workers 93 (17.4%). Fifty four patients (10.1%) were unemployed while 93 patients (17.4%) were homemakers (Table 1).

The majority of the affected population were from the Sudan mycetoma belt. There were 227 patients (42.6%) from Geizira State, 80 patients (15%) from Khartoum State, 62 patients (11.6%) from the White Nile State, 48 patients (9%) from the Sinner State and 44 patients (8.3%) were from Northern Kordofan State.

Most of the patients 390 (73.2%) had painless lesions. A history of trauma at the mycetoma site was recalled by only 104 patients (19.5%), while the majority 360 (67.5%) had no history of trauma and 61 patients (11.4%) were not sure of that. Concomitant medical problems were documented in only 18 patients (3.4%). Only 59 patients (11.1%) had a family history of mycetoma.

The study showed that, 359 patients (67.4%) had mycetoma of less than 5 years duration at presentation while only 48 patients had mycetoma for more than ten years. Most of the patients 277 (52%) had past surgery for mycetoma and recurrence and that ranged between once; 190 (35.6%), twice; 51(9.6%), thrice; 16(3.0%) and more than three times; 19 (3.9%).

The right hand was affected more frequently in this series, which was documented in 322 patients (60.4%) while the left one was affected in 211 patients (39.6%) (Table 1).

**Fig 1:**
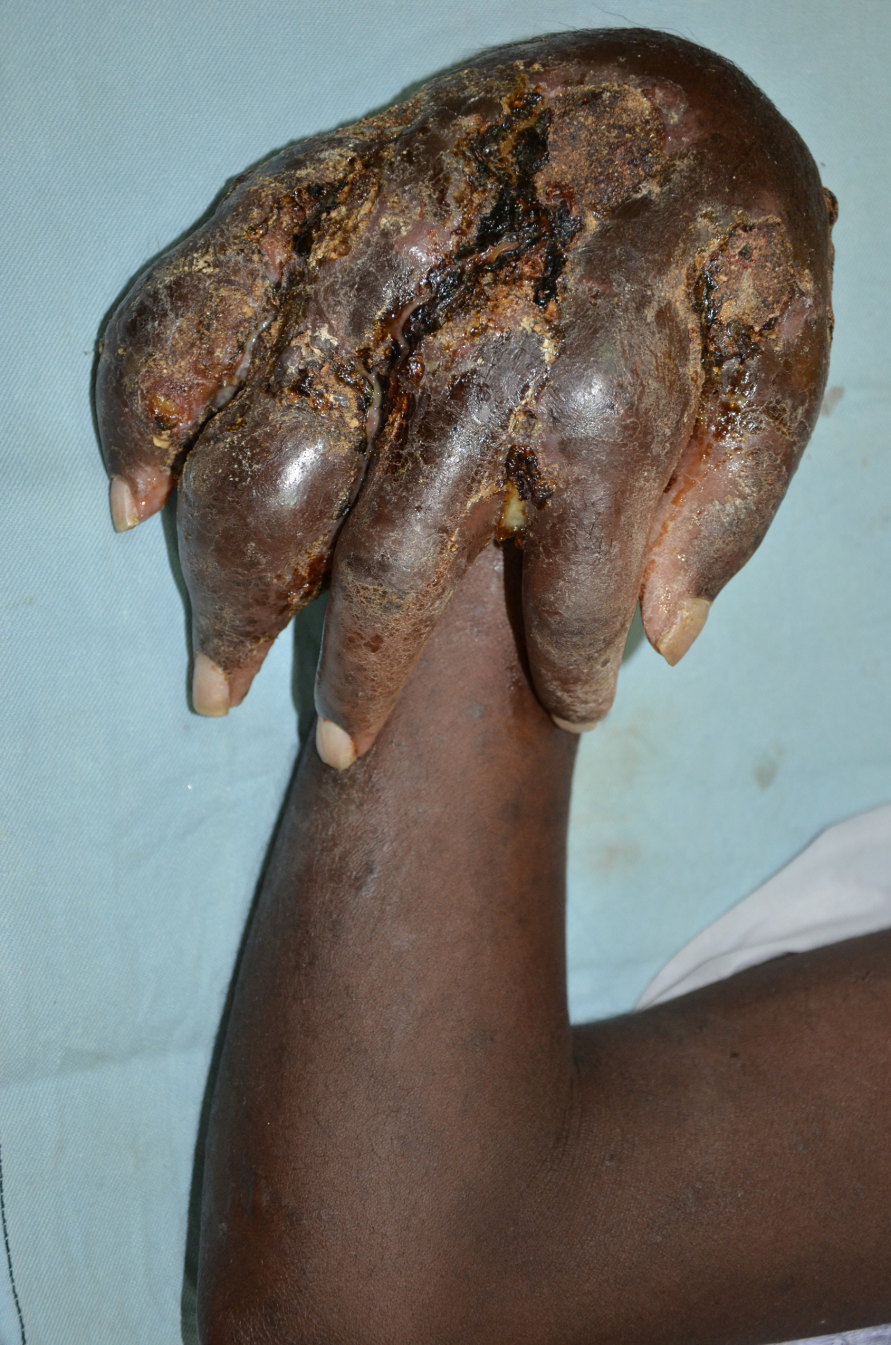
Showing massive hand actinomycetoma.

**Fig 2:**
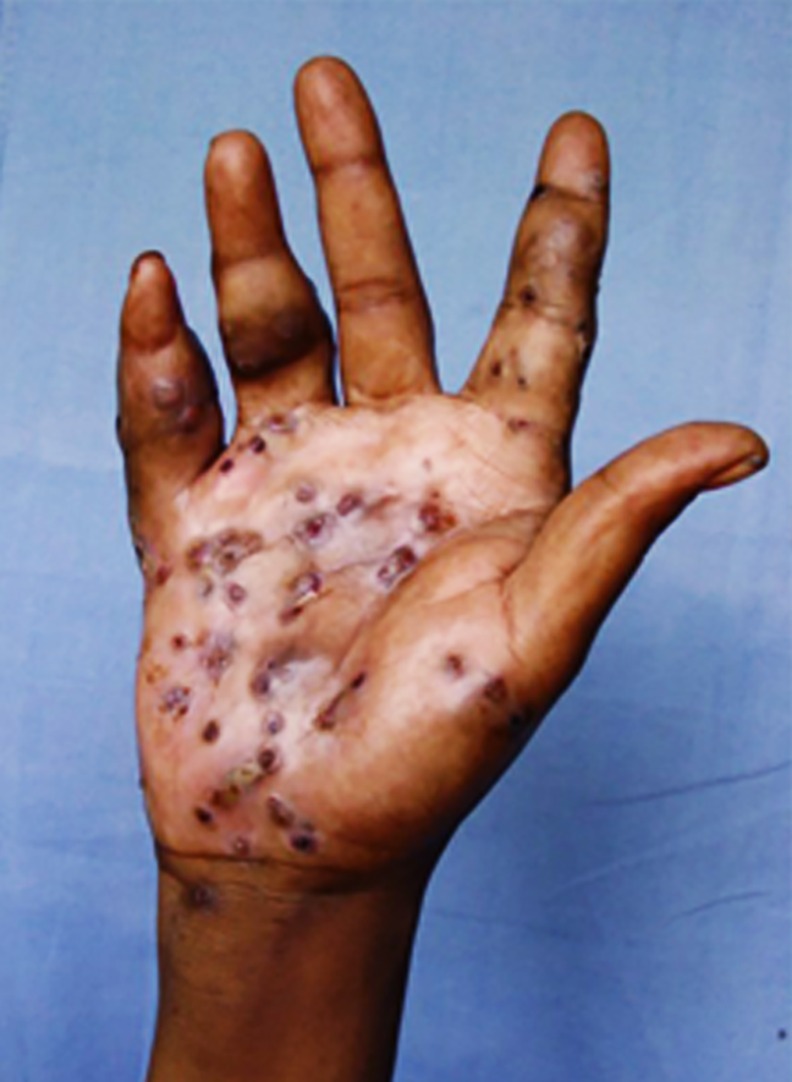
Showing massive eumycetoma with deformity.

The hand mycetoma lesions size at presentation were variables, they were classified as small lesions less 5 cm, moderate sized between 5–10 cm and massive lesions which were more than 10 cm in diameter. The study showed that, 245 patients (46%) had small lesions, 140 patients (26.1%) had moderate lesions and 122 patients (22.9%) had massive lesions, while 16 patients (3.0%) were operated upon recently elsewhere. All of the patients had mycetoma clinical triad, all had subcutaneous swelling, 80.7% had active or closed sinuses and 42.2% had discharge with grains (Figs 1 and 2).

The final diagnosis of mycetoma was achieved by a combination of at least two techniques and these techniques included X-ray examination, ultrasonic, cytological, histological techniques and grain culture.

**Fig 3:**
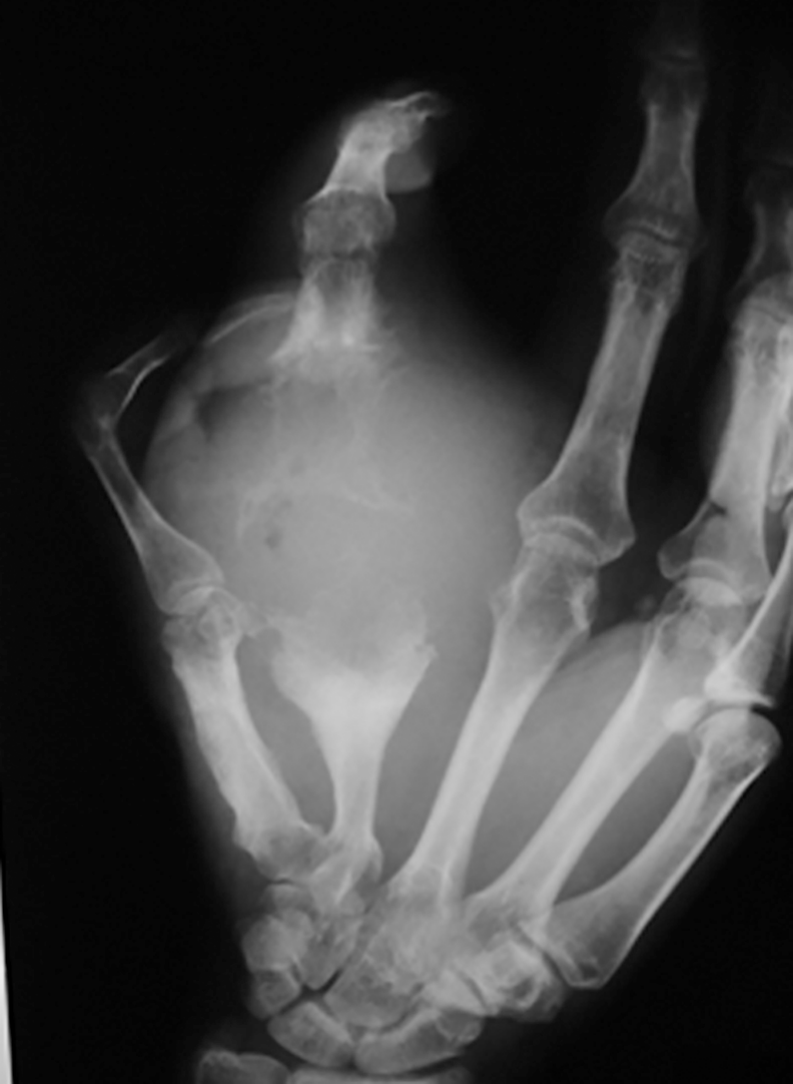
X-ray of right hand showing massive soft tissue mass, periosteal reaction and bone cavities in line with eumycetoma appearance.

X-ray examination of the affected series in two views showed, soft tissue swelling in 154 patients (28.9%), bone cavitation in 55 patients (10.3%), periosteal reaction in 11 patients (2.1%) and a combination of these findings in 41 patients (7.7%) and normal in 85 patients (15.9%) (Fig 3).

**Fig 4:**
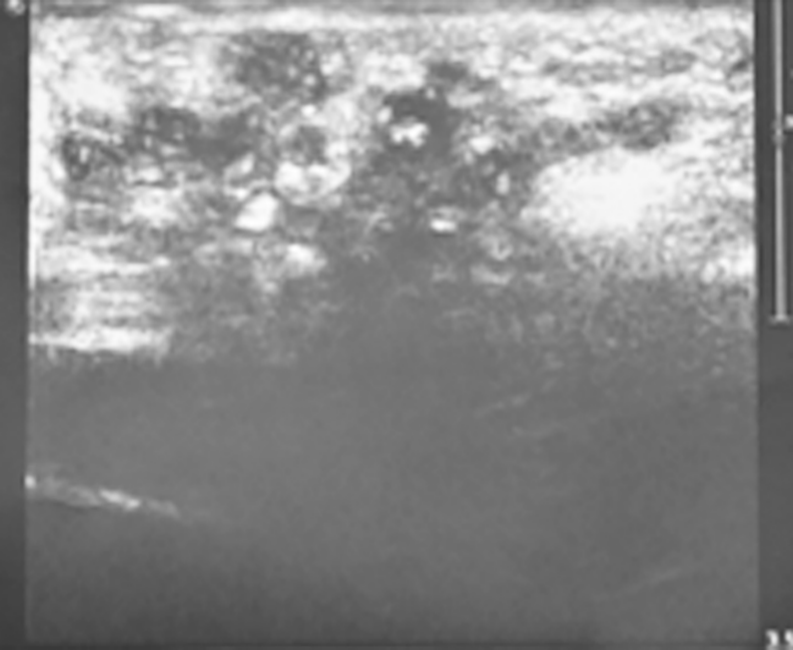
Ultrasound showing typical eumycetoma lesion; multiple thick walled cavities with sharp hyper-reflective echoes denoting grains without acoustic enhancement.

**Fig 5:**
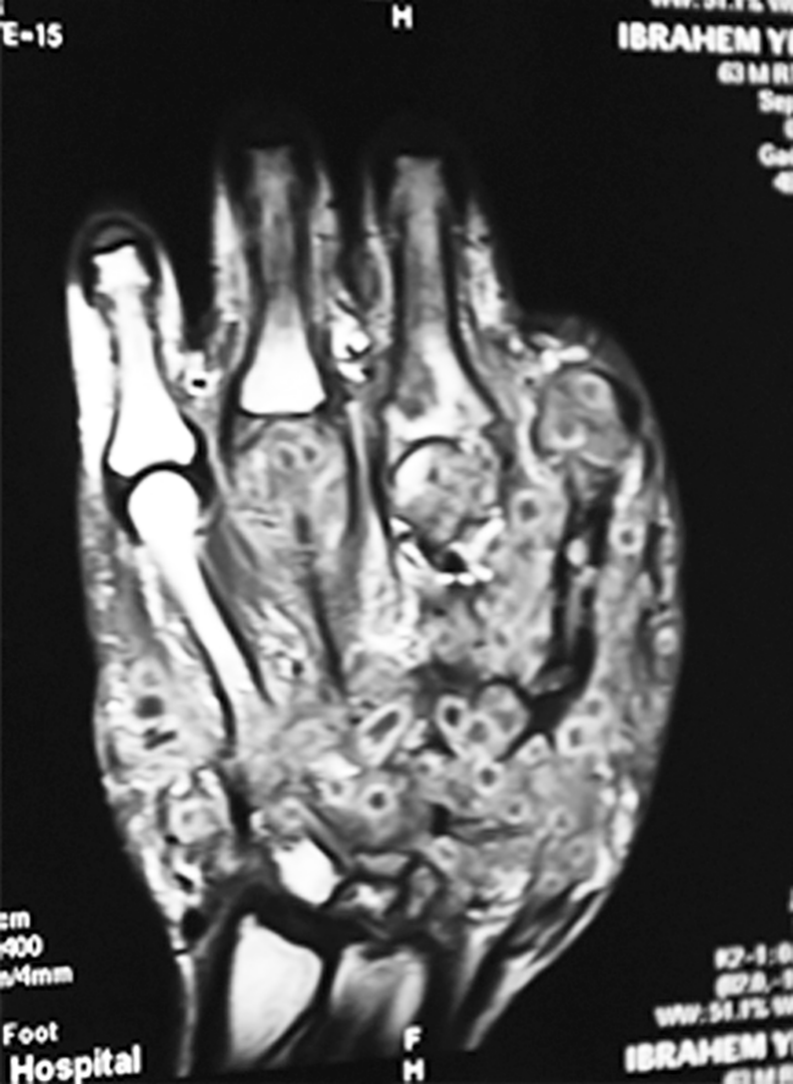
MRI showing involvement of soft tissue and bone by mycetoma and the dot-in-circle sign which is characteristic of for mycetoma.

In the present study, 199 patients underwent ultrasonography examination to establish the diagnosis of mycetoma and to differentiate between its two types. 165 patients (82.9%) had eumycetoma, 15 patients (7.5%) had actinomycetoma and in 19 patients (9.5%) no grains were detected and no definite diagnosis was established (Fig 4). Few patients had MRI examination which showed the typical mycetoma features (Fig 5).

In this series, 200 patients (37.5%) had histopathological examinations of surgical biopsies. The diagnosis of *M*. *mycetomatis* was established in 130 patients (65%), *Streptomyces somaliensis* in 29 patients (14.5%), *Actinomadura pelletierii* in five (2.5%) and *Actinomadura madurae* in four patients (2%). In seven patients (3.5%) grains were absent and the diagnosis was established by other modalities.

FNAC was performed in 258 patients (48.4%); 216 patients (83.7%) had *M*. *mycetomatis*, 20 patients (7.7%) had *Actinomadura madurae*, eight patients (3.1) had *Streptomyces somaliensis* and 14 patient (5.4%) no grains were detected.

**Fig 6:**
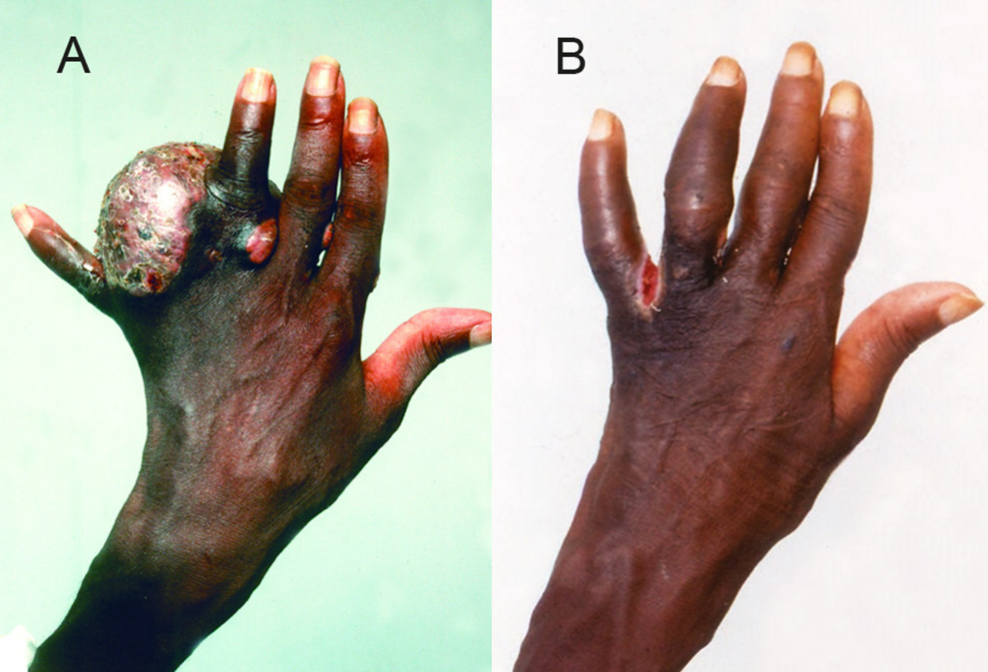
Showing massive hand actinomycetoma (A) pre and (B) post treatment with combination of Amikacin sulphate and co-trimoxazole.

Combination of antimicrobial agents was given for actinomycetoma which included streptomycin sulphate one gram daily and dapsone 100mg daily, or streptomycin and trimethoprim-sulfamethoxazole. More recently, trimethoprim-sulfamethoxazole 8/40 mg/kg/day combined with amikacin 15 mg/kg/day was given in the form of cycles (Fig 6) [16,17].

**Fig 7:**
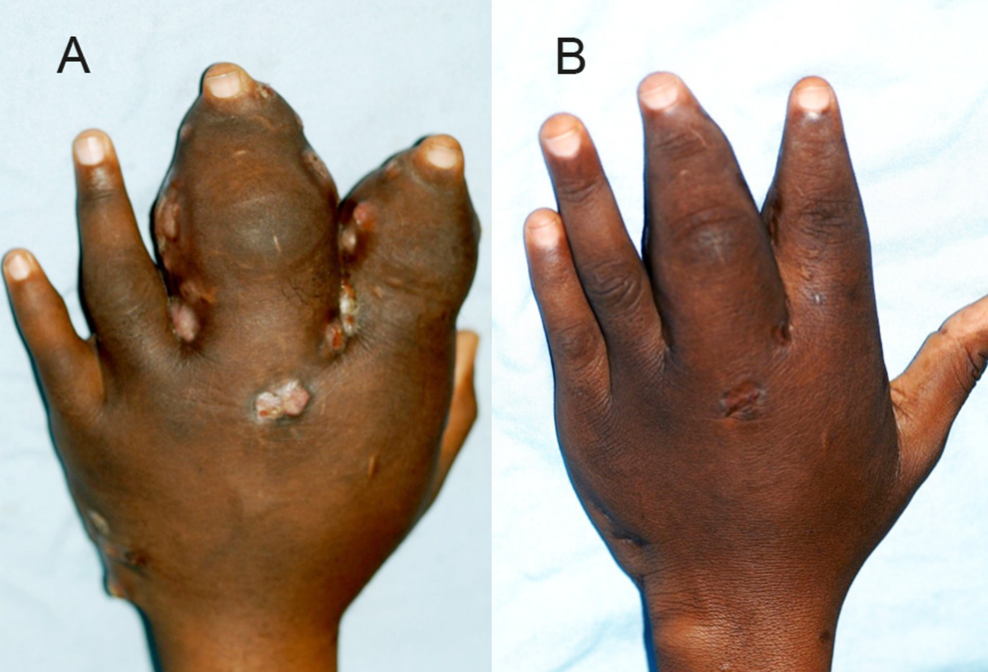
Showing hand eumycetoma (A) before surgery (B) after surgery.

A combination of various antifungal agents and different surgical procedures were offered to these patients. The antifungal agents prescribed were ketoconazole in a dose of 400–800 mg daily and 200–400 mg of itraconazole daily (Fig 7) [19,20].

The follow up and treatment dropout was 55% of patients which is rather high and cure was 25% of the regularly followed up patients. The reasons for this high dropout rate are multifactorial and that include the high treatment cost which is not affordable by the patients and their families, the treatment is prolong more than one year in most patients, the drugs in use are commonly toxic and have several side effects and the patients low health education and socio-economic status that made it difficult for them to travel to the center from their remote villages for treatment. All these can contribute to the poor treatment outcome.

## Discussion

This communication reports on 533 patients with hand mycetoma. It is considered the largest series ever reported and thus provides a significant addition to the literature. It confirms the previous reports that, the hand is the second frequent site for mycetoma [4, 9, 28]. The incidence of hand mycetoma reported here is in accordance with previous reports that ranged between 1.8% and 7.4%. [4, 13, 9, 28]. A report on mycetoma from a single centre in Mexico showed out of 482 patients, 36 patients (7.46%) had hand mycetoma [4]. In another series of 3933 patients from Mexico, most of the studied patients (60.29%) had extremities mycetoma [28].

Males were mostly affected in our series and this is in agreement with preceding reports from the Sudan and globally, however, the sex ratio reported in this series is smaller [1,2,4,22]. Research suggested sex hormones may play a role in this male predominance, but further studies are need to verify it [29].

The young adults were most frequent affected cohort in the present study which is in agreement with other series [1,2,4,22]. The affection of these young group is serious as they are the most active group in developing countries. It leads to serious socio-economic consequences and many of them drop out their education.

The study showed that, 76.4% of the studied population had no family history of mycetoma. The susceptibility to contract the disease can be due to environmental, genetic or immunogenic factors. However, susceptibility and resistance to mycetoma demands further in depth study.

Children and adolescents were the common affected group reported in this study, most of previous reports showed farmers and workers were affected most [1,2,4,28]. The explanation is indistinct but this may be due to fact that mycetoma is common in this age group, and children are commonly in contact with the soil during playing or helping their families in farming activities. As the soil may harbour the causative organisms and they are more prone to minor injuries which facilitate the inoculation of causative organism make them more liable to develop mycetoma.

Most of the affected patients 359 (67.4%) had disease duration of less than five years, which is rather short duration compared to previous reports [1, 2, 4, 9, 28]. This may be due to improvement in health education among the affected population and hand swelling may be recognised more frequent and earlier than other parts of the body. This contrast the long disease duration in patients with scrotum, gluteal region, vulva or perineum who are commonly reluctant to seek medical advice. This explanation may be supported be the fact that, 46% of the hand mycetoma patients reported with a small sized lesions at presentation.

The study showed that 52% of the studied population had past history of post- operative recurrence. This can be multifactorial and to mention but a few, surgery was performed in rural health centres, under local anaesthesia by inexperienced health assistants and in a poor surgical setting. Some patients had massive lesion with long disease duration that makes it difficult to completely excise all the affected tissues and hence the recurrence.

In agreement with the previously reported series [12], *Madurella mycetomatis* eumycetoma was the prevalent type in our series and this is due to the predominance of this organism in the Sudan. The other common causative organisms encountered were *Streptomyces somaliensis*, *Actinomadura madurae and Actinomadura pelletierii*.

Reviewing the medical literature revealed several hand mycetoma cases caused by other causative organisms. Pupaibul in 1982, reported hand mycetoma caused by *Phialophora Jeanselmei* [30] while *Leptosphaeria tompkinsii*, *black* grain causative organisms, was reported by Cartwright and colleagues [31] and by Machmachi and associates [32]. Hand mycetoma caused by *Nocardia caviae* was reported in few patients as well [33]. *Scedosporium boydii (Scedosporium apiospermum)* is a frequent cause of hand mycetoma. It was reported in a 75-year-old immunocompromised male patient who received long-term corticosteroid and immunosuppressant therapy for the treatment of nephrotic syndrome, in a patient with adult Still's disease and in a 52-year-old male heart transplant recipient [34, 35, 36]. *Scedosporium boydii* of the hand and forearm was reported in a patient with Behçet's disease treated with infliximab and chronic prednisone therapy [37]. A case of mycetoma caused by *Fusarium solani* with osteolytic lesions on the hand in a Brazilian farmer aged 71 years was reported [38]. The first case of hand *Arthrographis kalrae* eumycetoma which was cured by a 4-month course of itraconazole was reported in 1997 [39]. *Acremonium recifei* was reported to cause hand eumycetoma with white-yellowish grains mycetoma in immunocompetent patients [40, 41].

These organisms were not documented in this study and that may be due to the rarity of these organisms in the Sudan or they are under diagnosed. Hence, the use of PCR for organisms’ identification is required to identify such rare organisms and for planning the appropriate treatment.

The right hand was affected most (60.4%) and been the dominant hand in most of the patients, some researchers incline to attribute that to the causative organisms traumatic inoculation theory [1,2]. The clinical presentations of the studied patients were in line with previously reported. All had subcutaneous swelling, 80.7% had active or closed sinuses and 42.2% had discharge with grains, the mycetoma characteristic triad.

However, clinically hand mycetoma is to be differentiated from two other mycotic infections caused by a group of black fungi and these are phaeohyphomycosis and chromoblastomycosis [42,43]. Phaeohyphomycosis, a rare disease and sporadically reported is distinguished from mycetoma by the absence of grains formation while chromoblastomycosis is differentiated by the absence of sclerotic bodies. The black fungi are a group of fungi that are characterized by the development of a pale brown to black colour in the cell walls of their vegetative cells, conidia, or both. The differentiation between these three mycotic infections can be established by surgical biopsies and histopathological examination using special staining and by PCR identification.

Several radiological signs can be detected on conventional x-ray of the hand mycetoma [44]. In this study, fanning of the metacarpals bones, bone erosions, sclerosis, periostitis and soft tissue swelling were reported and the most common signs were soft tissue swelling (88%) and bones involvement (45%). These findings were seen in patients with advanced mycetoma. For this reason, more MRI and CT scans are currently in use to determine the disease extend along the different tissues planes. Mycetoma has distinctive MRI appearance, the “in-dot sign” is diagnostic. It can outline the skin, subcutaneous, muscles and bones involvement accurately and can grade the disease which help in patients’ management [45]. In mycetoma, the bone affection can accurately be assessed by CT scan.

In the present series it was difficult to determine the treatment outcome accurately and precisely due to high patients follow up dropout. The reasons for the high dropout rate are multifactorial and that include the patients’ dissatisfaction with the high cost and the prolonged treatment duration which is commonly more than one year, the drug side effects and complications, the patients low socio-economic status, the lack of health education and difficulty to reach the MRC, particularly during rainy seasons. All these had contributed to the poor treatment outcome also. To overcome these constrains and shortcomings a new treatment model was adopted by the MRC. The model consists of regular visits to endemic remote areas in the Sudan for early case finding and management. Through a mobile surgical teams, surgical treatment was carried at the local villages and recently free medicines were provided [47].

In conclusion, the hand is a precious human organ with complex functions and losing it through destruction, distortion, deformities, or amputation is unacceptable particularly due to benign inflammatory disease like mycetoma. Knowledge gaps in mycetoma epidemiology and pathogenesis will need to be addressed careful by further research, likewise novel diagnostic tools and treatment need be researched [46]. Until that time, health education and awareness are the cornerstone in prevention and reducing disease morbidity and mortality.

## Supporting Information

S1 Checklist STROBE Checklist. (DOC)
